# The Role of Epigenetics in Type 1 Diabetes

**DOI:** 10.1007/s11892-017-0916-x

**Published:** 2017-08-16

**Authors:** Samuel T. Jerram, Mary N. Dang, R. David Leslie

**Affiliations:** 1 Queen Mary University of London, Mile End Rd, London, E1 4NS UK; 2 The Blizard Institute, London, UK

**Keywords:** Diabetes, Type 1, Epigenetics, Methylation

## Abstract

**Purpose of Review:**

Epigenetics is defined as mitotically heritable changes in gene expression that do not directly alter the DNA sequence. By implication, such epigenetic changes are non-genetically determined, although they can be affected by inherited genetic variation. Extensive evidence indicates that autoimmune diseases including type 1 diabetes are determined by the interaction of genetic and non-genetic factors. Much is known of the genetic causes of these diseases, but the non-genetic effects are less clear-cut. Further, it remains unclear how they interact to cause the destructive autoimmune process. This review identifies the key issues in the genetic/non-genetic interaction, examining the most recent evidence of the role of non-genetic effects in the disease process, including the impact of epigenetic effects on key pathways.

**Recent Findings:**

Recent research indicates that these pathways likely involve immune effector cells both of the innate and adaptive immune response. Specifically, there is evidence of cell type-specific enrichment in altered DNA methylation, changes which were temporally stable and enriched at gene regulatory elements.

**Summary:**

Epigenomics remains in its infancy, and we anticipate further studies will define how the interaction of genetic and non-genetic effects induces tissue-specific destruction and enhances our ability to predict, and possibly even modify that process.

## Introduction

Epigenetics is defined as mitotically heritable changes in gene expression that do not directly alter the DNA sequence [1–3]. These changes are therefore seen as non-genetic factors that interact with genes, although they can be affected by inherited genetic variation. As regulators of transcription, epigenetic mechanisms play a necessary role in maintaining normal growth, development, differentiation and genome stability [4]. Genes play a major role in disease pathogenesis; however, the environment can modify this effect by introducing a complex interaction between the two. This review briefly discusses how non-genetic (environmental) factors could interact with that genetic risk to induce autoimmune diseases. Specifically, we discus type 1 diabetes (T1D) and the potential role of epigenetic effects in that process leading to autoimmune diabetes.

## Genetic Risk for Autoimmune Diseases

The destructive process causing T1D is thought to be immune-mediated with a potent autoimmune element involving altered adaptive immunity. There is strong evidence that the disease has an immunological basis given that the principle genetic susceptibility resides in immune response genes and that other ‘autoimmune’ diseases share the same or similar genetic risk factors. However, there may be more to that disease pathogenesis than an aggressive autoimmune response given that the insulin gene itself has been implicated in genetic risk, implying either the insulin-secreting cell itself or altered central tolerance through insulin gene expression in the thymus.

The heritability of a disease is an estimate of the genetic risk in the context of non-genetic predisposition. It is usually estimated in classic twin studies, and for a T1D identical twin the concordance rate (both twins affected), irrespective of age at diagnosis, is consistently less than 100%, which implies a non-genetically determined effect [5]. However, the concordance rate declines with age at diagnosis of the index twin, indicating that in adult-onset T1D the genetic impact is limited, and certainly lower than that in childhood-onset disease.

Genes associated with T1D are well-established and have four broad functions:

1. Antigen presentation and T cell repertoire formation;
2. Decreased T cell signalling/activation;
3. Increased T1 interferon and antiviral response;
4. Cytokine production/signalling [6]. However, T1D is unlikely to be a single disease since there is disease heterogeneity, notably in the rate of β cell loss, immunogenotypes, responsiveness to immunotherapies, and in islet pathology [7]. For example, multidimensional cluster analysis showed two equal-sized patient agglomerations in childhood-onset T1D characterised by pro-inflammatory (IFN-γ-positive, multi-autoantibody-positive) and partially regulated (interleukin-10-positive, pauci-autoantibody-positive) responses [8]. In addition, the appearance of autoantibodies close to birth in at risk children indicates two clusters: one associated with very early induction at age 9 months involving insulin autoantibodies and IA-2 autoantibodies with histocompatibility (HLA) DR4 disease risk, the other involving the later induction of glutamic acid decarboxylase autoantibodies (GADA) with HLA DR3 disease risk [9, 10]. The immunopathological processes (endotypes) which underlie T1D risk could be distinct given the different age at disease induction and the differences in disease onset, with the former being diagnosed in early childhood and the latter often substantially later [8]. Some patients, likely more than 50%, present in adult life without an immediate need for insulin therapy, that is with adult-onset non-insulin requiring diabetes. However, a substantial proportion of the latter have multiple diabetes-associated autoantibodies and HLA genetic heterozygosity, so the disease heterogeneity is more likely a spectrum of features, such as age at diagnosis, and not distinct clusters of different disease endotypes [11].

The mechanism whereby HLA genes are associated with autoimmune diseases has only recently become apparent. There are strong associations in trans between variation in the HLA locus and T cell receptor (TCR) V gene usage. Fine-mapping of that association identified specific amino acids from MHC genes that biased V gene usage. Thus, in the T cell receptor (TCR)–peptide–MHC complex, MHC variants, some linked to autoimmune diseases, can directly affect the TCR–MHC interaction illustrating trans-effects mediated by protein–protein interactions consistent with intrinsic TCR–MHC specificity [12]. Since HLA alleles can be protective for T1D and other autoimmune diseases (e.g. the kidney disease Goodpasture’s disease and multiple sclerosis), it is not surprising that the protective effect operates in the same format. HLA-DR15 confers a markedly increased disease risk to Goodpasture’s disease; the protective HLA-DR1 allele is dominantly protective in trans with HLA-DR15 [13]. T cells autoreactive to a key renal antigen, collagen α3135–145, are expanded in patients with Goodpasture’s disease. HLA-DR15 and HLA-DR1 exhibit distinct peptide repertoires and binding preferences and present the α3135–145 epitope in different binding registers. T cells in HLA-DR15 transgenic mice exhibit a conventional T cell phenotype that secretes pro-inflammatory cytokines. In contrast, HLA-DR1-α3135–145 tetramer + T cells in HLA-DR1 and HLA-DR15/DR1 transgenic mice are predominantly CD4 + Foxp3 + regulatory T cells (Treg cells) expressing tolerogenic cytokines. Patients with Goodpasture’s disease display a clonally expanded α3135–145-specific CD4+ T cell repertoire [13]. Accordingly, there is now a mechanistic basis for the dominantly protective effect of HLA and the HLA susceptibility in autoimmune disease, whereby HLA polymorphism shapes the relative abundance of self-epitope specific cytotoxic T cells or Treg cells that leads to protection or causation of autoimmunity. A similar HLA DR3/DR4 (susceptibility) and HLA DR 15 (protection) pattern can be seen in type 1 diabetes.

## Non-Genetic Effects Causing T1D

There is now substantial evidence that non-genetic factors play a role in the development of autoimmune diseases. The appearance of altered diabetes-associated autoantibodies in early childhood implies a role for these non-genetic, likely environmental, effects at that stage [9, 10, 14]. Environmental factors that have been implicated in the aetiology of autoimmune diseases include the following: temperate climate, increased hygiene, decreased rates of infection, vaccinations and antibiotics, drugs (methyl donors such as hydralazine), wheat consumption, iodine levels, cigarette smoking and increasing wealth (possibly all relevant for most autoimmune and atopic diseases). Such non-genetic effects have also been implicated for T1D, including: gross national product, overcrowding in childhood and virus infections, early exposure to cow’s milk, reduced rates or duration of breast feeding and vitamin D and nitrite consumption [15] The clear evidence that genetic effects cause T1D should be compared with the many hypotheses for the nature of the non-genetic effects and the clear inference that we do not know the precise identity of those non-genetic effects.

### Disease Incidence

Autoimmune diseases have become more prevalent in the last century [16]; the incidence of T1D has even increased several-fold in the last 30 years [17]-a timeframe which rules out genetic evolution. In addition, studies of the incidence of T1D in migrant populations have shown a convergence towards the risk of the host population [18, 19], including amongst migrant groups from regions of the world with low incidence. The greater heritability of T1D in childhood is also consistent with a more potent genetic effect on immune responses in children. Brodin and colleagues, using classic twin studies, measured 204 different parameters, including cell population frequencies, cytokine responses and serum proteins [20]. They found that 77% of these responses are dominated (> 50% of variance) and 58% almost completely determined (> 80% of variance) by non-heritable influences. In addition, some of these parameters become more variable with increasing age, suggesting the cumulative influence of environmental exposure. Firm evidence for a non-genetic-genetic interaction comes from evidence that there is an increased risk of rheumatoid arthritis in subjects who both smoke and carry the HLA-DR4 risk allele [21]. In addition, a viral role in T1D is supported by the observation that four rare variants of interferon-producing genes are associated with a reduced risk of developing T1D [22]. Each variant was associated with a 50% reduction in risk. All four variants occur in the same gene, IFIH, which affects the expression and structure of its protein IFIH1, a helicase enzyme. These genes are part of the interferon regulatory 7 factor inflammatory network in monocytes and macrophages, which are antiviral in function [22].

### Diet

There is contradictory evidence for a relationship between diet and development of T1D. Exposure to breast milk and cows’ milk in infancy has not been found to impact risk of developing islet cell autoantibodies or T1D in most prospective birth cohort studies [23–26], although the ABIS study in Sweden did report such an effect [27]. The relationship between introduction of solid foods in infancy, and in particular cereals, is also unclear, with timing of introduction thought to play a role. The DAISY cohort study identified a U-shaped association between timing of introduction of cereal (not limited to gluten containing), and development of islet autoimmunity [25], while in BABYDIAB the association was only seen with early exposure to gluten [26] and in ABIS with late introduction of gluten [28].

### Infections

While it is possible that bacterial infection may play a role in development of pancreatic inflammation, the main infectious culprits in the development of T1D are thought to be viral, with enteroviruses firmly in the frame in both animal [29] and human studies [30–33]. Intriguingly, a possible mechanism for enterovirus persistence has been identified in the context of enteroviral myocarditis [34, 35]. Ideally, viral remnants would be found in the islet of patients with T1D but not in the non-diabetic population but that critical evidence is currently lacking.

### Hygiene Hypothesis

Alongside the theory of viral infection as an environmental trigger for islet cell autoimmunity, the hygiene hypothesis implicates the reduction in childhood illness due to improved hygiene in the rise of autoimmune diseases [16]. In the main, large-scale prospective cohort studies have failed to identify a link between reduced early childhood infections and development of islet autoimmunity [36], though they have demonstrated an increase in islet cell autoimmunity following more frequent maternal infections in pregnancy and upper respiratory viral infections in childhood [37]. Similarly, owning pets in infancy reduce the risk of asthma with a 13% reduction in a substantial Swedish study [38]. An extension of the hygiene hypothesis proposes that it is not a reduction in childhood infections that is the culprit, but a reduction in herd immunity, notably to enterovirus infection amongst pregnant women resulting from increased hygiene, which in turn increases foetuses’ and new-borns’ exposure to the virus [39]. This theory finds limited support from animal studies [40].

### Intestinal Microbiota

The nature of some of the environmental factors linked to the development of T1D, such as early childhood diet as described above, caesarean delivery and use of antibiotics, implicate the human microbiome, and in particular its development in early childhood. Animal studies have clearly shown that changes in the microbiome impact the innate immune response and predispose to autoimmune diabetes, potentially through toll-like receptors [41]. In man, the data is less clear with small-scale studies describing a lower microbial diversity, including a reduction in butyrate-producing and mucin-degrading bacteria, in children demonstrating autoimmunity to islet cells [42], although larger more comprehensive and prospective studies are required before a clear decision is made on this relationship. While it is difficult to draw firm conclusions from the data on non-genetic factors in the development of T1D, it is clear that T1D cannot be explained simply by genetics alone, even allowing for the established heterogeneity of disease. Non-genetic factors undoubtedly play a role, and as further research in the field is undertaken the exact nature of this role should become clearer.

## Genetic and Non-genetic Interaction Causing Autoimmune Diseases

**Fig. 1:**
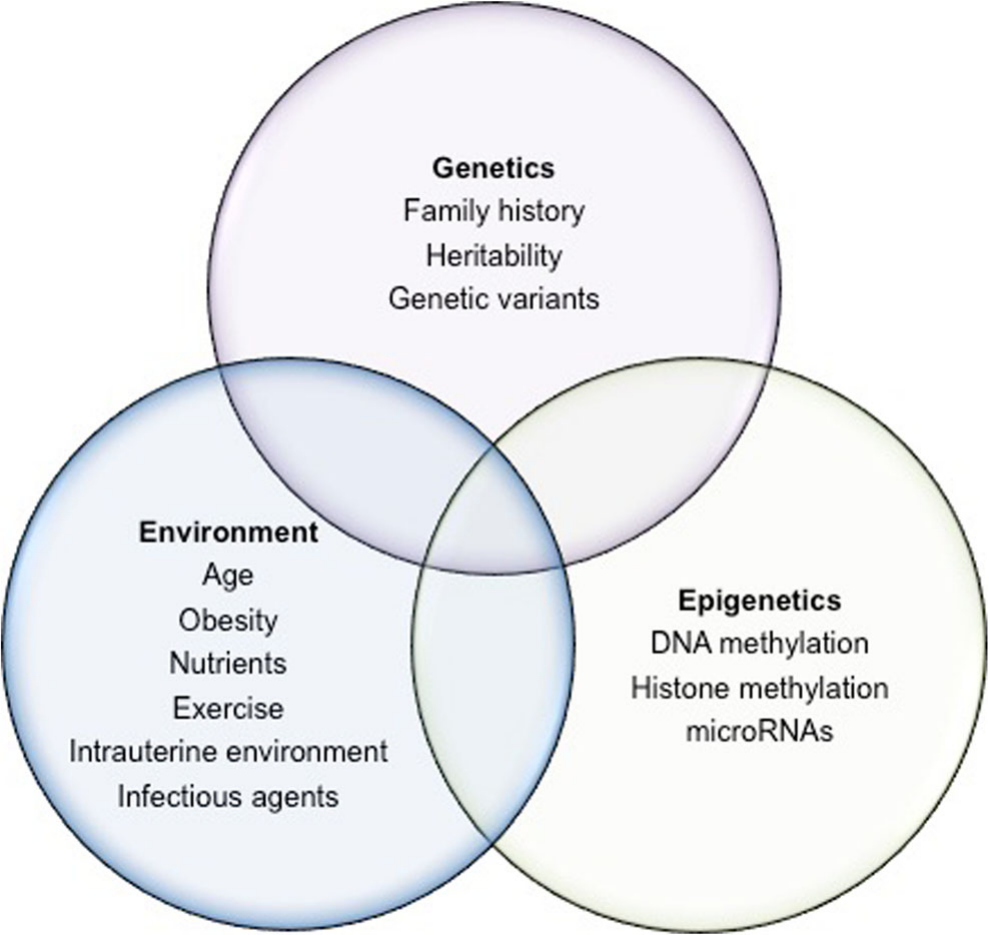
Interaction between genes, environment and epigenetics in disease. The genome can give rise to many phenotypes. Although genetics, epigenetics and the environment can affect phenotype outcomes independently, it is the complex interaction that gives rise to diseases such as T1D. Evidence for this includes MZ twin studies in which disease concordance was not 100%. Factors such as age and dietary nutrients have been shown to affect the epigenome and some of these epigenetic changes can occur in utero

Given that both genetic and non-genetic effects interact to induce most common complex diseases, the same is likely to be true for T1D (Fig. 1). We have already alluded to the increased risk of rheumatoid arthritis in subjects who both smoke and carry the HLA-DR4 risk allele [21]. In addition, there is evidence to implicate genes in the interferon regulatory 7 factor inflammatory antiviral immune cell network [22]. Exposure to the anti-hypertensive drug hydralazine can induce systemic lupus erythematosus in patients who are slow acetylators (a genetic determined trait), female and with the HLA-DR4 genotype [43]. As hydralazine is a demethylating agent, it might be envisaged that the side-effect results directly or indirectly from changes in DNA methylation. That non-genetic effects can interact with genetic factors through epigenetics to cause autoimmunity comes from the study of rheumatoid arthritis. Meng and colleagues found that DNA methylation of the CpG promoter site cg21325723 can mediate the gene-environment interaction between rheumatoid-associated single nucleotide polymorphism rs6933349 and smoking [21]. This interaction, impacting the risk of developing the rheumatoid arthritis (RA)-associated citrullinated peptide autoantibodies, has been duplicated in both Caucasian and Asian populations [21]. By implication, genetic and non-genetic factors can initiate an autoimmune process that is associated with autoimmune disease, and that interaction can result from altered gene expression linked to epigenetic changes.

## Epigenetic Architecture

**Fig. 2:**
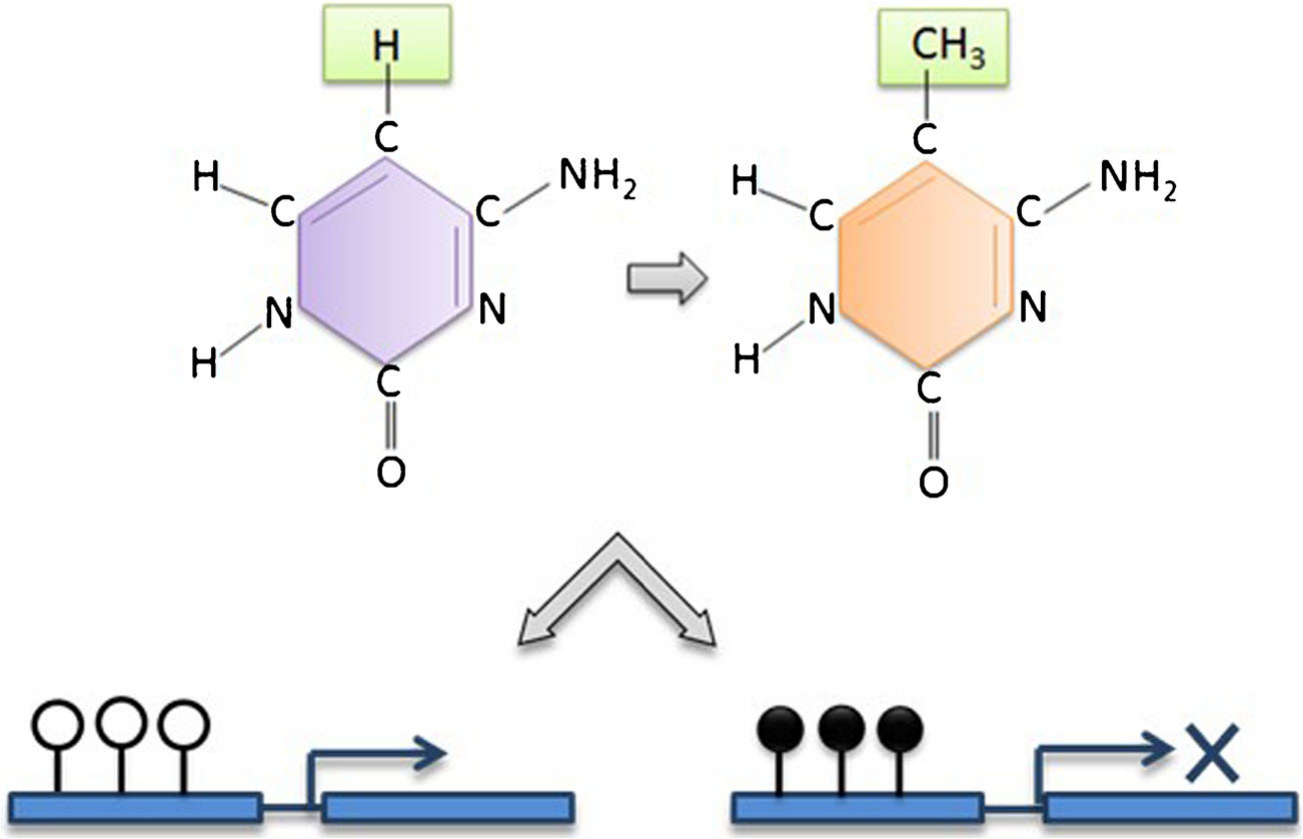
DNA methylation and gene expression. A simplified schematic of DNA methylation and its effect on gene expression. DNA methylation occurs by the covalent addition of a methyl group to the 5′ carbon of a cytosine nucleotide. Methylated CpG sites (*filled lollipop*) are associated with gene silencing, whereas unmethylated sites (*unfilled lollipop*) are associated with transcriptional activity. In the case of hydroxymethylation, at the 5′ carbon position, the hydrogen molecule is replaced by a hydroxymethyl group

**Fig. 3:**
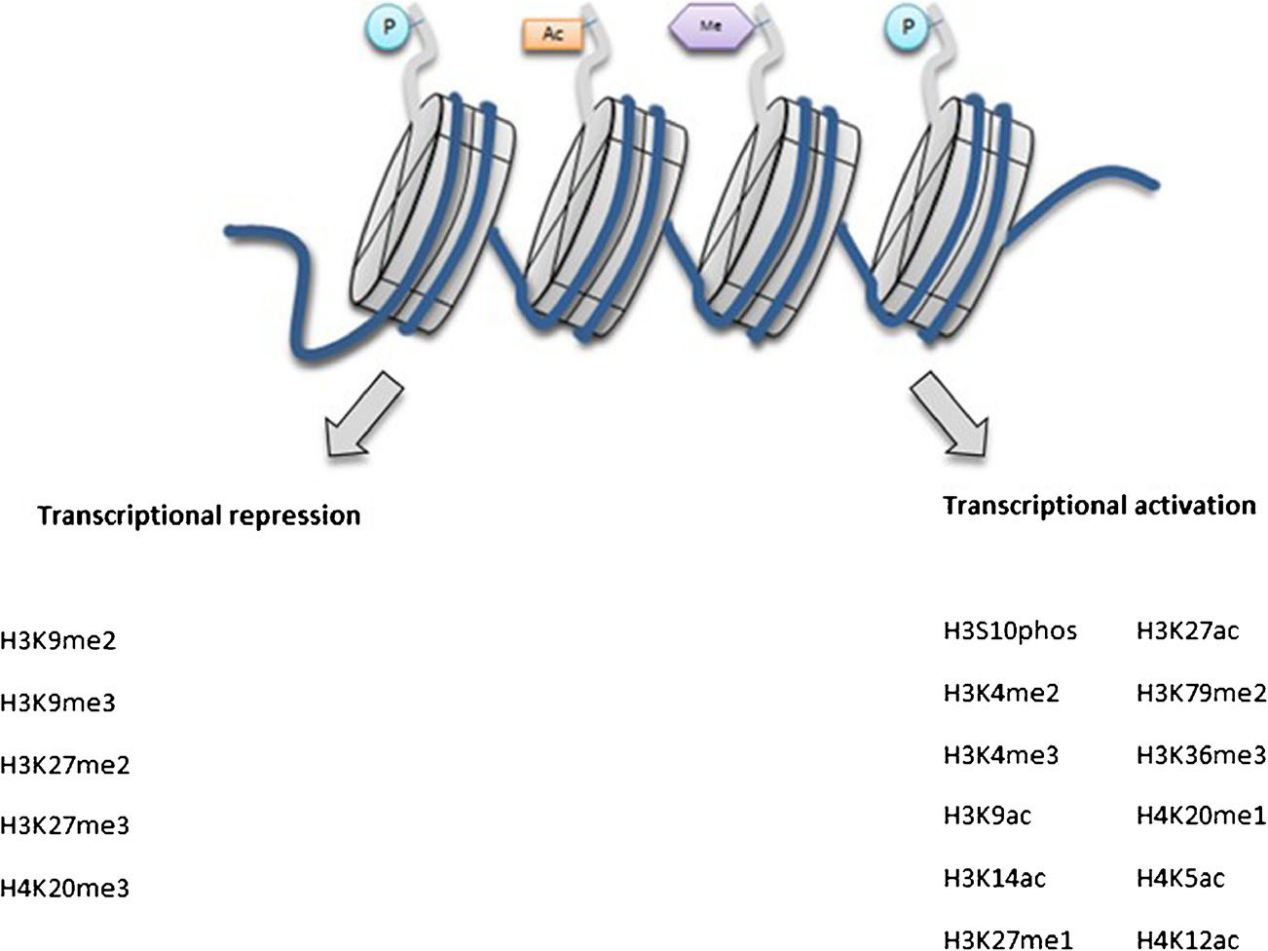
Chromatin structure with histone modifications. Simplified schematic of the chromatin structure with histone modifications. Different modifications result in conformational changes to the chromatin. The nucleosome is made up of two copies of each histones H2A, H2B, H3 and H4, wrapped by 146 base pairs of DNA. Methylation of H3 on lysine at position 4, 36 and 79 leads to an actively transcribed open chromatin structure. Methylation at H3 on lysine at positions 9 and 27 leads to transcriptional repression. *P* phosphorylation, *Ac* acetylation, *Me* methylation

Epi- is the Ancient Greek term for ‘above’ as in ‘epiphenomenon’. Thus, epigenetics is the study of the marks ‘above’ the genes, that is the chemical tags (including methylation and acetylation) on DNA and RNA. The best-characterised epigenetic modifications or marks are DNA methylation, histone post-translational modifications and non-coding RNA-mediated gene silencing [44]. DNA methylation, which involves the covalent addition of a methyl group to the 5′ carbon at a CpG site, is associated with gene silencing (Fig. 2), and has been reported to be essential for embryonic development [45], genomic imprinting [46, 47] and X-inactivation in mammals [48, 49]. Histone post-translational modifications are alterations in the chromatin structure affecting the expression and repression of genes by enzymatic modification of core histones [50] (Fig. 3). The enzymes that are involved in this process are known as histone acetyltransferase (HAT) and histone deacetylases (HDACs). Lastly, gene expression can be regulated by non-coding RNAs (ncRNAs), which are short RNA transcripts which include miRNAs and siRNAs [51, 52].

There are different regulatory mechanisms that act at different levels to remodel the chromatin structure. Alongside histone modifications and transcription factors, several cis-regulatory elements, including enhancers, promoters, silencers and insulators, are crucial to the function of the genome. An enhancer is a region of DNA that enhances transcription levels of a gene through the binding of transcription factors [53]. There are more than a million enhancers; therefore, many more than there are genes, so that a number of genes are regulated by the same enhancer, which may co-localise with CpGs [54, 55]. Gene enhancers can be found upstream or downstream of genes and do not necessarily act on the closest promoter, i.e. they can act as distant promoters. In order to do this, the DNA loops around, bringing the specific promoter to the initiation complex; this enhancer-promoter loop has approximately 120 kilobases [56]. Enhancers may be accompanied by insulators, which are located between the enhancers and promoters of adjacent genes and can limit phenotypic gene expression despite genetic activation [53].

Genetic activation occurs following binding of transcription factors to DNA. A section of DNA is made available to transcription factors binding by unwinding of the chromatin with reduced nucleosome density and low DNA methylation making available selected sites to cleavage by DNase enzymes (DNase hypersensitivity sites or DHSs) [57, 58]. DHSs represent regions of transcriptionally active genome, and there were approximately 2.9 million such DHSs identified by the Encyclopaedia of DNA Elements (ENCODE) Consortium [57]. Given the interaction between regulatory elements and epigenetic marks, regulation of gene expression is far more complex than initially envisaged.

Although the genetic components of autoimmune diseases have been discussed extensively, the current effort to narrow down the determining role of different environmental factors in the disease process is a more difficult task. Evidence suggests that through the complex interplay of genetic and environmental factors, epigenetics plays a role in the development of autoimmune diseases.

## Epigenetics of Autoimmune Diseases

The fact that autoimmune disease concordance rates in monozygotic twins are consistently less than 100% (range 13–61%) [59–61], coupled with evidence of epigenetic modifications of gene expression, highlights the need to further define the role of external factors and how they influence gene expression in autoimmune diseases. Below, we discuss epigenetic regulation in autoimmune diseases including RA, systemic lupus erythematosus and autoimmune thyroid diseases, before considering T1D.

### Rheumatoid Arthritis

Rheumatoid arthritis is a chronic autoimmune disease that affects the joints [62, 63]. Epigenetic modifications associated with RA include methylation changes in T and B cells [64–66] and synovial fibroblasts [67–69]. We have already noted that DNA methylation of the CpG promoter site cg21325723 can mediate the gene-environment interaction between RA-associated single nucleotide polymorphism rs6933349 and smoking [21]. In another study, a discovery cohort of 50 participants with RA was compared to 75 controls [65]. The authors had found differential methylation in 64 CpGs in B cells using the Illumina HumanMethylation450 BeadChip. This observation was validated in an independent cohort of patients. In RA synovial tissues, the balance between HATs and HDACs activity is strongly shifted towards HAT activity, consistent with histone hyperacetylation [70]; hyperacetylation of histone H3 in the IL-6 promoter was shown to increase IL-6 expression by RA synovial fibroblasts [71]. A number of subtypes of ncRNAs have been associated with RA such as miR-146, miR-155 and miR-223, as described by Kolarz et al. [72].

### Systemic Lupus Erythematosus

Systemic lupus erythematosus (SLE) is an autoimmune disease characterised by acute and chronic inflammation that targets several tissues in the body. Similar to RA, studies into epigenetic dysfunctions have focused on immune cells due to their vital role in the pathogenesis of autoimmune diseases [73]. Recently, neutrophils and granulocytes from patients with SLE were found to be globally hypomethylated particularly in the interferon genes, MX1 and IFI44L [74]. Hypomethylation was also associated with interferon signalling in a study investigating epigenetic aberrations in CD4+ T cells, CD19+ B cells and CD14+ monocytes from participants with SLE compared to healthy controls [75]. Histone modifications have been identified in SLE; for example, higher methylation in the HDAC6 promoter led to lower HDAC6 mRNA expression in persons with SLE than in controls [76]. Several miRNAs have been shown directly or indirectly to be involved with DNMT1 by means of methylation of certain genes in CD4+ T cells [77, 78]. One study in particular observed that miR-125a expression was reduced in participants with SLE leading to elevated expression of RANTES [79]. This observation is supported by increased serum levels of RANTES in patients with SLE [80]. Expression of miR-146a was increased in peripheral blood mononuclear cells and synoviocytes in patients with SLE [81, 82], whereas downregulation of miR-200a-3p was involved in the hypoproduction of IL-2 by T cells [83].

### Autoimmune Thyroid Diseases

Two main autoimmune thyroid diseases are Hashimoto’s thyroiditis and Graves’ disease; both are characterised by the loss of immunological self-tolerance [84, 85]. Hashimoto’s thyroiditis involves a cell-mediated autoimmune destruction of the thyroid leading to hypothyroidism, while Graves’ disease is characterised by specific autoantibodies to the thyroid stimulation hormone receptor leading to hyperthyroidism [86]. In autoimmune thyroid disease patients, differentially methylated regions have been identified from genomic DNA. There were also differences in DNA methylation between patients with Graves’ and a control group in ICAM1, a gene involved in cell antigen processing and presentation [87]. Additionally, in participants with Graves’ disease, sorted CD4+ and CD8+ T cells were analysed for H3K4me3 and H3K27ac histone marks [88]. The authors observed hypermethylation in genes involved in T cell signalling with decreased levels of H3K4me3 and H3K27ac histone marks in T cell signalling genes. An epigenetic process known as X chromosome inactivation has also been associated with autoimmune thyroid diseases [89]. X chromosome inactivation is an essential epigenetic event in female embryonic development that leads to transcriptional silencing of one of the X chromosomes. Brix et al. [90] found skewed X chromosome inactivation in female twins with Graves’ disease and Hashimoto’s thyroiditis, an observation supported by another group who studied a larger patient cohort [91].

## Epigenetic Effects and T1D Risk

It can be envisaged that epigenetic marks illustrate a process whereby non-genetic effects can alter gene transcription and translation. In a simplistic understanding of epigenetics, it might be said that epigenetics explains the difference between your ear and your nose (which have identical DNA) as it does the differences between an MZ twin pair (who also have identical DNA).

Several features suggest T1D could be subject to epigenetic effects. (1) MZ twins have a high discordance rate especially when they are aged more than 15 years at diagnosis; (2) The risk of T1D has increased in recent years more rapidly than could be accounted for by genetic changes alone; (3) the risk for the offspring of a father with T1D is more than that risk for a mother with T1D (6 vs. 1% respectively). (4) HLA haplotype sharing does not account for diabetes risk; instead, it is age at diabetes onset which determines that risk. To illustrate the last point, a study of extended HLA haplotypes determined in 2134 siblings from the Bart’s-Oxford Study that age of the proband at diagnosis, but not HLA haplotype sharing, was an independent determinant of sibling risk. By implication, non-HLA genes or epigenetic/environmental factors that accelerate the progression of T1D in the proband strongly affect risk in siblings [92].

### DNA Methylation and Diabetes Risk

Direct studies of epigenetic changes have implicated changes associated with insulin secretion and diabetes risk [93]. A genome-wide DNA methylation quantitative trait locus (mQTL) analysis in human pancreatic islets was performed using 574,553 SNPs with genome-wide DNA methylation data of 468,787 CpG sites targeting 99% of RefSeq genes in islets from 89 donors [93]. The authors found 383 CpG sites (0.08% of tested CpGs), showing significant associations after correction for multiple testing including known diabetes loci, e.g. *ADCY5*, *KCNJ11*, *HLA-DQA1*, *INS*, *PDX1* and *GRB10*. CpGs of significant cis-mQTLs were overrepresented in the gene body and outside of CpG islands. Causal inference tests identified SNP-CpG pairs with DNA methylation in human islets as potential mediators of the genetic association with gene expression or insulin secretion. Functional analyses further demonstrated that identified candidate genes (*GPX7*, *GSTT1* and *SNX19*) directly affect key biological processes such as proliferation and apoptosis in pancreatic β cells. The study showed that genome-wide genetic and epigenetic variation can interact to influence gene expression, islet function and potential diabetes risk in humans [93]. Support for the role of epigenetic modification of DNA by methylation can also be found in mouse models [94].

### DNA Methylation and Autoimmunity

The problem with these studies of epigenetics is their limited power because of the enormous numbers of CpG sites identified. Using a dense genotyping of autoimmune disease it was found that T1D is more similar genetically to other autoantibody-positive diseases, most particularly to juvenile idiopathic arthritis, while least significantly close to ulcerative colitis [95••]. T1D SNPs localise to enhancer sequences in thymus, T and B cells, and CD34+ stem cells, and this observation illustrates the power of epigenetic analysis to identify those cells which are actively using the genes associated with a given tissue, given that all cells contain every gene—a state that genetics alone cannot resolve [95••]. Recent epigenetic studies used arrays such as 450 K by Illumina in which the distal enhancer regions were excluded. Bisulphite sequencing (very expensive) and the new arrays can now define enhancer DNA methylation. The importance of this shortfall is evident with some of the earlier studies as evidence from those indicated that the causal variants of T1D were found in open-reading frames in gene enhancer regions of CD4, CD8 and CD14 cells [96].

Epigenetic readers are proteins that recognise histone modifications and facilitate code-based transcriptional programming. Bromodomain- and plant homeodomain (PHD)-containing proteins can serve as readers of acetylation and methylation on histones, respectively. A recent study examined the function of SP140, a bromodomain- and PHD-containing reader in immune cells, and found that SP140 plays an essential role in repressing lineage-inappropriate genes in macrophages. Since functionally impaired SP140 polymorphisms are associated with Crohn’s disease, epigenetic readers could regulate immune responses in normal and diseased states.

Given the evidence that DNA methylation of the promoter regions is strongly inherited (with very high concordance rates between identical twins), it follows that twin pairs discordant for a disease are the perfect test-bed to analyse epigenetic differences that contribute to that disease. We performed an epigenome-wide association study in 52 monozygotic twin pairs discordant for T1D in three highly selected immune effector cell types [97•]. The immune cells were CD4+ T cells, CD19+ B cells and CD14 + CD16– monocytes and we interrogated DNA before and after bisulphite conversion from these cells using the Illumina 450 K array noted above [97•]. Bisulphite converts unmethylated cytosine to uracil, while methylated cytosine is protected from the conversion; thus, it is possible to identify DNA methylation using these arrays. By using disease-discordant monozygotic twins, our strategy reduced major confounding effects, such as inter-individual genetic variability and in utero effects and substantially reduced the differential impact of shared childhood environmental factors. There was a remarkable concordance between twins of each pair consistent with a strong shared genetic/non-genetic effect on CpG methylation in DNA promoter regions. QQ plot for the identification of differentially methylated CpG positions (DMPs) between T1D-discordant MZ twin pairs in different immune effector cell types showed that only the DMP cg01674036 reached genome-wide significance in T cells and a mean DNA methylation difference of 2.3% [96]. In contrast, a QQ plot for the identification of differentially variable methylated CpG positions (DVPs) between the MZ twin pairs found striking hypervariability in all cell types, particularly pronounced in B cells [96]. Compared to the healthy, unrelated individuals, patients with T1D showed cell type-specific enrichment with changes, which were temporally stable and enriched at gene regulatory elements. These cell type-specific gene regulatory circuits identified pathways involved in immune cell metabolism and the cell cycle, notably including mTOR signalling. It seems likely that the DVPs emerged after birth as they were not detected in cord blood of new-borns who later developed clinical T1D, though there remains the potential for cord blood to detect better defined epigenetic changes [98]. These results could implicate epigenetic changes that could contribute to disease pathogenesis. But equally, the epigenetic changes could be secondary to the diabetes process and not disease risk or alternatively that the changes could impact disease outcome. Future studies of subjects with prediabetes using arrays interrogating distal enhancer regions should be more informative and are underway. 

If genetic and epigenetic analyses are to have clinical utility, it will likely be in disease prediction, disease outcome prediction and prediction of best therapeutic approaches. Certainly, the mTOR pathway has been implicated in the development of diabetes-associated damage [99]. If changes are predictive of altered diabetes metabolism or of diabetes-associated complications then their clinical utility would be as valuable in autoimmune T1D as in type 2 diabetes [100]. For example, blood-based epigenetic biomarkers reflecting age-related DNA methylation changes in human islets, e.g. KLF14, FHL2, ZNF518B and FAM123C, have been associated with both insulin secretion and type 2 diabetes [101].

### Epigenetics and Vascular Risk

Hyperglycaemia increases the risk of development and progression of vascular complications related to diabetes. Such effects could be due to epigenetic changes involving altered DNA methylation, histone modification and changes in miRNA. Certainly, epigenetic modifications mediated by histone methyltransferases are associated with gene-activating events including enhanced expression of pro-inflammatory networks implicated in vascular injury [101–103]. A genetic variation in a gene coding for a histone methyltransferase is potentially protective for a diabetic microvascular complication; a minor T allele of the exonic SNP rs17353856 in the SUV39H2 was associated with diabetic retinopathy (genotypic odds ratio 0.75) [103]. These networks could involve innate immune effects represented by the active transcription of the NFkappaB-p65 genes, itself linked with persisting epigenetic marks such as enhanced H3K4 and reduced H3K9 methylation [103]. In addition, the let-7 miRNA family plays a key role in modulating inflammatory responses critical to the pathogenesis of atherosclerosis [104]. There is evidence that changes in glycaemia-associated epigenetic DNA methylation persist for several years [105•].

## Conclusion

If genetic and epigenetic analyses are to have clinical utility, it will likely be in disease prediction, disease outcome prediction and prediction of best therapeutic approaches. Epigenetic effects represent one molecular mechanism whereby genetic and non-genetic factors can interact. The evidence outlined here suggests that epigenetic factors play a role in many aspects of the pathogenesis of autoimmune diseases including, potentially, the pathogenesis of long-term consequences of diabetes.
